# Alterations of consciousness and mystical-type experiences after acute LSD in humans

**DOI:** 10.1007/s00213-016-4453-0

**Published:** 2016-10-07

**Authors:** Matthias E. Liechti, Patrick C. Dolder, Yasmin Schmid

**Affiliations:** Psychopharmacology Research, Division of Clinical Pharmacology and Toxicology, Department of Biomedicine and Department of Clinical Research, University Hospital Basel, University of Basel, Hebelstrasse 2, CH-4031 Basel, Switzerland

**Keywords:** LSD, Altered states of consciousness, Mystical experiences

## Abstract

**Rationale:**

Lysergic acid diethylamide (LSD) is used recreationally and in clinical research. Acute mystical-type experiences that are acutely induced by hallucinogens are thought to contribute to their potential therapeutic effects. However, no data have been reported on LSD-induced mystical experiences and their relationship to alterations of consciousness. Additionally, LSD dose- and concentration-response functions with regard to alterations of consciousness are lacking.

**Methods:**

We conducted two placebo-controlled, double-blind, cross-over studies using oral administration of 100 and 200 μg LSD in 24 and 16 subjects, respectively. Acute effects of LSD were assessed using the 5 Dimensions of Altered States of Consciousness (5D-ASC) scale after both doses and the Mystical Experience Questionnaire (MEQ) after 200 μg.

**Results:**

On the MEQ, 200 μg LSD induced mystical experiences that were comparable to those in patients who underwent LSD-assisted psychotherapy but were fewer than those reported for psilocybin in healthy subjects or patients. On the 5D-ASC scale, LSD produced higher ratings of blissful state, insightfulness, and changed meaning of percepts after 200 μg compared with 100 μg. Plasma levels of LSD were not positively correlated with its effects, with the exception of ego dissolution at 100 μg.

**Conclusions:**

Mystical-type experiences were infrequent after LSD, possibly because of the set and setting used in the present study. LSD may produce greater or different alterations of consciousness at 200 μg (i.e., a dose that is currently used in psychotherapy in Switzerland) compared with 100 μg (i.e., a dose used in imaging studies). Ego dissolution may reflect plasma levels of LSD, whereas more robustly induced effects of LSD may not result in such associations.

**Electronic supplementary material:**

The online version of this article (doi:10.1007/s00213-016-4453-0) contains supplementary material, which is available to authorized users.

## Introduction

Lysergic acid diethylamide (LSD) is the prototypical hallucinogen (Nichols 2016; Passie et al. 2008). LSD became famous, with a high cultural influence, in the 1960s. LSD continues to be used for recreational and personal purposes (Krebs and Johansen 2013). Additionally, there is much interest in its therapeutic potential (Baumeister et al. 2014; Davenport 2016; Gasser et al. 2014; Gasser et al. 2015; Krebs and Johansen 2012; Kupferschmidt 2014). Only one modern study has tested the therapeutic effects of LSD in patients (Gasser et al. 2014), whereas several clinical trials have recently evaluated the therapeutic potential of psilocybin (Bogenschutz et al. 2015; Carhart-Harris et al. 2016a; Garcia-Romeu et al. 2015; Griffiths 2016; Grob et al. 2011; Guss 2016), a similar serotonergic hallucinogen (Rickli et al. 2016). A series of studies showed that psilocybin acutely induced mystical experiences in healthy subjects and patients (Garcia-Romeu et al. 2015; Griffiths et al. 2008; Griffiths et al. 2011; Griffiths et al. 2006; MacLean et al. 2011). Additionally, greater acute effects of psilocybin on the Mystical Experience Questionnaire (MEQ; Barrett et al. 2015; Griffiths et al. 2006; MacLean et al. 2012) were associated with positive long-term effects on mood and personality in healthy subjects (Griffiths et al. 2008; Griffiths et al. 2011; Griffiths et al. 2006; MacLean et al. 2011) and better therapeutic outcomes in patients with anxiety, depression, and substance use disorder (Garcia-Romeu et al. 2015; Griffiths 2016; Griffiths et al. 2008; Griffiths et al. 2011; Griffiths et al. 2006; MacLean et al. 2011). Early studies reported on mystical experiences after experimental administration of LSD, but methodological details are missing (Turek et al. 1974). Whether and the extent to which LSD produces mystical-type effects in the MEQ are currently unknown. Therefore, we characterized the effects of 200 μg LSD on the MEQ and evaluated the way in which mystical experiences are related to LSD-induced increases in 5 Dimensions of Altered States of Consciousness (5D-ASC) scale scores and plasma levels of LSD.

Clinical experimental research with LSD has recently seen a resurgence (Carhart-Harris et al. 2016b; Carhart-Harris et al. 2015; Carhart-Harris et al. 2016c; Dolder et al. 2015b; Dolder et al. 2016; Kaelen et al. 2015; Kaelen et al. 2016; Lebedev et al. 2016; Roseman et al. 2016; Schmid et al. 2015; Speth et al. 2016; Strajhar et al. 2016; Tagliazucchi et al. 2016; Terhune et al. 2016). An increasing amount of data has been generated on the effects of LSD (75 μg) on various neuronal correlates of brain activation (Carhart-Harris et al. 2016c; Kaelen et al. 2016; Lebedev et al. 2016; Roseman et al. 2016). Researchers have correlated subjective drug effects with brain functional magnetic resonance imaging (fMRI) data (Carhart-Harris et al. 2016c; Kaelen et al. 2016; Lebedev et al. 2016; Roseman et al. 2016). This approach likely produces significant findings for subjective effects that show large between-subject variance but not for subjective effects of the substance that are consistently present in all subjects. Lower doses of LSD may also result in more variable responses across subjects compared with higher doses. Furthermore, higher doses of LSD (e.g., 200 μg) that are currently used therapeutically (Gasser et al. 2014) may produce more pronounced but also qualitatively different subjective effects (Dolder et al. 2016). Importantly, plasma concentrations of LSD have not been determined in any of the published LSD fMRI studies to date; therefore, unclear is the way in which LSD exposure in the body is linked to subjective effects in these studies. Therefore, a second goal of the present study was to describe the subjective peak effects of two doses of LSD (100 and 200 μg) using the 5D-ASC scale (Studerus et al. 2010). The 5D-ASC scale has been used in all of the recent experimental studies with LSD (Carhart-Harris et al. 2016b; Carhart-Harris et al. 2016c; Schmid et al. 2015; Tagliazucchi et al. 2016) and with many other psychedelics, providing an opportunity to compare findings between studies and across substances and research groups. Thus, the present study assessed LSD dose- and plasma concentration-response functions using the 5D-ASC scale in 40 subjects (Dolder et al. 2015b; Dolder et al. 2016; Schmid et al. 2015), thus allowing comparisons with other studies that used the 5D-ASC scale but did not determine plasma LSD concentrations (Carhart-Harris et al. 2016b; Carhart-Harris et al. 2016c; Kaelen et al. 2016; Lebedev et al. 2016; Roseman et al. 2016; Speth et al. 2016; Tagliazucchi et al. 2016; Terhune et al. 2016). A third goal of the present study was to assess associations across subjects between the peak and total plasma exposure to LSD and its effects on 5D-ASC scale scores (Studerus et al. 2010). The effects of 100 μg LSD on 5D-ASC scale scores are reported for the first time in the present study, whereas the effects of 200 μg have been previously published (Schmid et al. 2015). However, the latter study did not evaluate dose- or concentration-response functions. Other data that were generated in the present study have been previously reported including acute and subacute adverse effects (Dolder et al. 2015b; Dolder et al. 2016; Schmid et al. 2015; Strajhar et al. 2016).

## Material and methods

### Study design

We performed two similar studies using double-blind, placebo-controlled, cross-over designs with two experimental test sessions (LSD and placebo) in a balanced order. Study 1 used a dose of 100 μg LSD and placebo in 24 subjects. Study 2 used 200 μg LSD and placebo in 16 subjects. The washout periods between sessions were at least 7 days. The studies were conducted in accordance with the Declaration of Helsinki and approved by the local ethics committee. The administration of LSD to healthy subjects was authorized by the Swiss Federal Office for Public Health, Bern, Switzerland. All of the subjects provided written consent before participating in either of the studies, and they were paid for their participation. The studies were registered at ClinicalTrials.gov (NCT02308969, NCT01878942).

### Participants

Forty healthy participants were recruited from the University of Basel campus via online advertisement. Twenty-four subjects (12 men, 12 women; 33 ± 11 years old [mean ± SD]; range, 25–60 years) participated in study 1, and 16 subjects (8 men, 8 women; 29 ± 6 years old; range, 25–51 years) participated in study 2. The inclusion and exclusion criteria were identical for both studies. Subjects younger than 25 years of age were excluded from participating in the study. Additional exclusion criteria were age >65 years, pregnancy (urine pregnancy test at screening and before each test session), personal or family (first-degree relative) history of major psychiatric disorders (assessed by the semi-structured clinical interview for *Diagnostic and Statistical Manual of Mental Disorders*, 4th edition, Axis I disorders by the study physician and an additional interview by a trained psychiatrist), use of medications that may interfere with the study medication, chronic or acute physical illness (abnormal physical exam, electrocardiogram, or hematological and chemical blood analyses), tobacco smoking (>10 cigarettes/day), lifetime prevalence of illicit drug use >10 times (except for tetrahydrocannabinol), illicit drug use within the last 2 months, and illicit drug use during the study (determined by urine drug tests). The subjects were asked to abstain from excessive alcohol consumption between test sessions and particularly limit their use to one standard drink on the day before the test sessions. Additionally, the participants were not allowed to drink xanthine-containing liquids after midnight before the study day. Eleven subjects had used a hallucinogen, including LSD (six participants), one to three times, and most of the subjects (29) were hallucinogen-naive. We performed urine drug tests at screening and before each test session, and no substances were detected during the study.

### Study procedures

Each study included a screening visit, a psychiatric interview, two 25-h experimental sessions, and an end-of-study visit. The experimental sessions were conducted in a quiet standard hospital patient room. The participants were resting in hospital beds except when going to the restroom. Only one research subject and one or two investigators were present during the experimental sessions. The participants could interact with the investigator, rest quietly, and/or listen to music via headphones, but no other entertainment was provided. LSD or placebo was administered at 9:00 AM. The subjects were never alone during the first 12 h after drug administration, and the investigator was in a room next to the subject for up to 24 h while the subjects were asleep (mostly from 1:00 AM to 8:00 AM).

### Study drug

LSD (*d*-LSD hydrate, HPLC purity >99 %, Lipomed AG, Arlesheim, Switzerland) was administered in single oral doses of 100 or 200 μg as gelatin capsules. Note that these LSD hydrate doses correspond to LSD tartrate doses of 123 and 246 μg, respectively. In the 1960–1970s, small doses of LSD tartrate of 25–150 μg were typically used in “psycholytic therapy” and higher doses of >200 μg in “psychedelic” therapy (Pahnke et al. 1970). The dose used in a recent LSD-assisted psychotherapy study was 200 μg LSD hydrate (Gasser et al. 2014). Both doses used in the present study were within the range of doses that are taken for recreational purposes (Passie et al. 2008). Corresponding placebo capsules were used.

### Measures

#### Mystical-type experiences

In study 2, mystical experiences were assessed using a German version (Supplementary Appendix 1) of the 43-item MEQ (Griffiths et al. 2006; MacLean et al. 2012; Pahnke 1969) embedded in the 100-item States of Consciousness Questionnaire (SOCQ; (Griffiths et al. 2006). The original English questionnaire was independently forward-translated into German by two translators with German as their mother tongue. Discrepancies between the two forward-translated versions and a previous German version were then discussed and selected items backtranslated. The version was then pretested for comprehension by persons with previous LSD or MDMA use.

The MEQ has been used in numerous experimental and therapeutic trials with psilocybin (Garcia-Romeu et al. 2015; Griffiths et al. 2008; Griffiths et al. 2011; Griffiths et al. 2006; MacLean et al. 2011). The MEQ items provide scale scores for each of seven domains of mystical experiences: internal unity, external unity, sacredness, noetic quality (as real as or more real than everyday reality), deeply felt positive mood, transcendence of time and space, and ineffability/paradoxicality (difficulty describing the experience in words). The total of all scale scores was used as an overall measure of the mystical-type experience. We also derived the four scale scores of the newly validated revised 30-item MEQ: mystical, positive mood, transcendence of time and space, and ineffability (Barrett et al. 2015). A complete mystical experience was defined as scores ≥60 % on all MEQ30 factors (Barrett et al. 2015). The MEQ was administered 24 h after drug administration, and the participants were asked to retrospectively rate drug effects during peak drug effects. For comparison, we included MEQ ratings that were obtained 6 h after administration of 3,4-methylenedioxymethamphetamine (MDMA) and methylphenidate in another study using a similar research setting (Schmid et al. 2014). Additionally, we included MEQ ratings from patients who were treated with 200 μg LSD for anxiety related to life-threatening illness in another study (Diesch 2015; Gasser et al. 2014; Gasser et al. 2015). All of these additional MEQ findings have not been previously published in scientific journals and were obtained in studies that were previously described in detail (Diesch 2015; Gasser et al. 2014; Gasser et al. 2015; Schmid et al. 2014).

#### Alterations of consciousness

The 5D-ASC scale was used in both studies to assess the overall peak alterations of consciousness. The 5D-ASC scale measures altered states of consciousness and contains 94 items (visual analog scales). The instrument consists of five subscales/dimensions (Dittrich 1998) and 11 lower-order scales (Studerus et al. 2010). The 5D-ASC dimension “Oceanic Boundlessness” (27 items) measures derealization and depersonalization associated with positive emotional states, ranging from heightened mood to euphoric exaltation. The corresponding lower-order scales include “experience of unity,” “spiritual experience,” “blissful state,” and “insightfulness.” The dimension “Anxious Ego Dissolution” (21 items) summarizes ego disintegration and loss of self-control phenomena associated with anxiety. The corresponding lower-order scales include “disembodiment,” “impaired control of cognition,” and “anxiety.” The dimension “Visionary Restructuralization” (18 items) consists of the lower-order scales “complex imagery,” “elementary imagery,” “audio-visual synesthesia,” and “changed meaning of percepts.” Two additional dimensions describe “Auditory Alterations” (15 items) and “Reduction of Vigilance” (12 items). The scale is well-validated and widely used to characterize the subjective effects of various psychedelic drugs (Carhart-Harris et al. 2016b; Hasler et al. 2004; Hysek et al. 2011; Schmid et al. 2015; Vollenweider et al. 2007; Vollenweider and Kometer 2010). In addition to the subscale analyses, we also analyzed the effects on ego dissolution item 71 (the boundaries between myself and my surroundings seemed to blur) because the concept of ego dissolution was often used in recent imaging studies (Tagliazucchi et al. 2016). The 5D-ASC scale was administered 24 h after drug administration, and the participants were asked to retrospectively rate the drug effects. 5D-ASC ratings were also performed at 3 and 10 h in study 1.

### Analysis of plasma LSD concentrations

Blood was collected into lithium heparin tubes before and 0.5, 1, 1.5, 2.5, 3, 4, 6, 8, 10, 12, 16, and 24 h after LSD administration. The 0.5, 1.5, and 2.5 h samples were not collected in study 1. Blood samples were immediately centrifuged, and the plasma was rapidly stored at −20 °C and later analyzed using liquid-chromatography-tandem mass-spectrometry as previously reported (Dolder et al. 2015a; Steuer et al. 2016). Maximal plasma concentrations (*C*_max_) and total exposure (area under the plasma concentration-time curve [AUC]) were estimated using compartmental modeling in Phoenix WinNonlin 6.4 (Certara, Princeton, NJ, USA). A one-compartment model was used with first-order input, first-order elimination, and no lag time.

### Statistical analyses

The data analysis was performed using Statistica 12 software (StatSoft, Tulsa, OK, USA). Differences between LSD and placebo or between the 100 and 200 μg doses of LSD were compared using dependent or independent *t* tests, respectively. Associations between outcome measures were assessed using Pearson correlations. Significance was assumed at *p* < 0.05.

## Results

### Mystical-type experiences

**Fig. 1:**
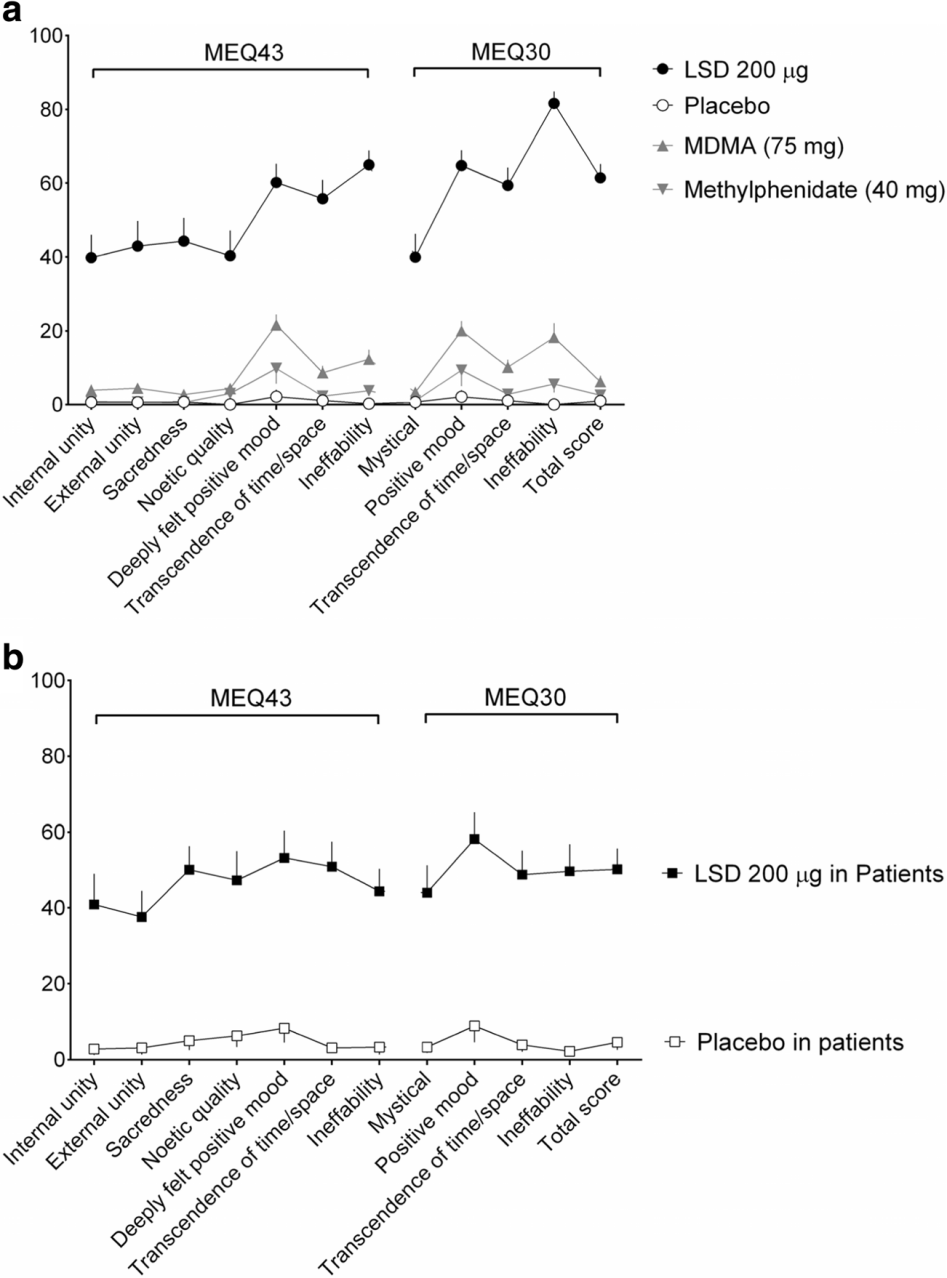
Effects of LSD on the Mystical Experience Questionnaire (MEQ). **a** In the present study in healthy subjects, LSD (200 μg) significantly increased scores on all scales of the MEQ43 and MEQ30 compared with placebo (Table 1). The data are expressed as the mean ± SEM in 16 subjects. For comparison, 3,4-methylenedioxymethamphetamine (MDMA; 75 mg) and methylphenidate (40 mg) produced small increases in MEQ ratings in 30 different participants in another study in the same research setting (Schmid et al. 2014). **b** Effects of LSD on the MEQ in patients with anxiety in the context of life-threatening illness. The data were analyzed identically to the data that were obtained in the present study. The study and patient characteristics have been previously published in detail (Diesch 2015; Gasser et al. 2014; Gasser et al. 2015; Schmid et al. 2014). Similar to the present study, the MEQ was administered on the day after LSD (200 μg) or active placebo (25 μg LSD) administration and was embedded into the larger 100-item States of Consciousness Questionnaire (SOCQ; Griffiths et al. 2006). The patient data are expressed as the mean ± SEM in 11 subjects for LSD (200 μg, same formulation as in the present study) and four subjects for placebo. On the 43- and 30-item versions of the MEQ, LSD (200 μg) increased MEQ rating scores in the patients in the therapeutic setting **(b)** to a similar extent as in the healthy subjects in the present study **(a)**. Notably, the placebo response (a very low dose of LSD of 25 μg was used as the active placebo) in the patients was small **(b)**, which was also similar to the response in healthy subjects in the present study **(a)**

LSD (200 μg) significantly increased all MEQ scores compared with placebo (Fig. 1a, Table 1). The effects of MDMA and methylphenidate on MEQ scores are included for comparison (Fig. 1a). The effects of LSD (200 μg) and placebo on MEQ scores in 11 patients during LSD-assisted psychotherapy (Gasser et al. 2014) are also shown in Fig. 1b. LSD-induced mystical experiences were comparable in healthy subjects in the laboratory setting in the present study and in patients in the therapeutic setting (Fig. 1b). Only two subjects in each of the studies had a complete mystical experience. The MEQ30 total scores were <5 % in both settings after placebo administration (Fig. 1b).

**Table 1:** Statistics for the effects of LSD in the 5D-ASC and MEQ

	LSD 100 μg *T* test vs. placebo		LSD 200 μg *T* test vs. placebo		LSD 100 vs. 200 μg *T* test	
	*T*=	*P=*	*T*=	*P=*	*T*=	*P=*
5 Dimensions Altered States of Consciousness (ASC) scale						
Total ASC score	9.72	<0.001	10.02	<0.001	2.23	<0.05
Oceanic boundlessness	8.44	<0.001	9.61	<0.001	1.89	NS
Anxious ego dissolution	6.43	<0.001	4.01	<0.001	1.50	NS
Visionary restructuralization	9.79	<0.001	15.32	<0.001	2.34	<0.05
Auditory alterations	3.72	<0.01	5.87	<0.001	0.42	NS
Reductions of vigilance	7.44	<0.001	5.93	<0.001	0.79	NS
Experience of unity	6.85	<0.001	7.77	<0.001	0.68	NS
Spiritual experience	4.31	<0.001	3.91	<0.001	1.10	NS
Blissful state	6.56	<0.001	8.27	<0.001	3.00	<0.01
Insightfulness	4.11	<0.001	5.81	<0.001	2.28	<0.05
Disembodiment	6.93	<0.001	5.87	<0.001	0.13	NS
Impaired control and cognition	7.01	<0.001	5.04	<0.001	0.86	NS
Anxiety	3.02	<0.001	2.04	NS	1.37	NS
Complex imagery	7.10	<0.001	7.48	<0.001	0.31	NS
Elementary imagery	9.96	<0.001	11.12	<0.001	0.57	NS
Audio-visual synsthesia	9.19	<0.001	12.52	<0.001	1.96	NS
Changed meaning of percepts	6.25	<0.001	9.66	<0.001	3.39	<0.01
Ego dissolution (item 71)	7.63	<0.001	5.32	<0.001	0.36	NS
Mystical Effects Questionnaire (MEC43)						
Internal unity	NA	NA	6.22	<0.001	NA	NA
External unity	NA	NA	6.08	<0.001	NA	NA
Sacredness	NA	NA	6.80	<0.001	NA	NA
Noetic quality	NA	NA	5.71	<0.001	NA	NA
Deeply felt positive mood	NA	NA	11.43	<0.001	NA	NA
Transcendence of time/space	NA	NA	10.63	<0.001	NA	NA
Ineffability	NA	NA	16.22	<0.001	NA	NA
Mystical Effects Questionnaire (MEQ30)						
Mystical	NA	NA	5.99	<0.001	NA	NA
Positive mood	NA	NA	13.13	<0.001	NA	NA
Transcendence of time/space	NA	NA	11.12	<0.001	NA	NA
Ineffability	NA	NA	25.14	<0.001	NA	NA
MEC30 total score	NA	NA	14.91	<0.001	NA	NA

Sixteen subjects participated in the high-dose study (200 μg) and 24 subjects in the moderate-dose study (100 μg). Dependent *T* tests were performed to assess differences from placebo, and independent *T* tests were performed to assess differences between doses of LSD

*NA* not assessed

### Alterations of consciousness

**Fig. 2:**
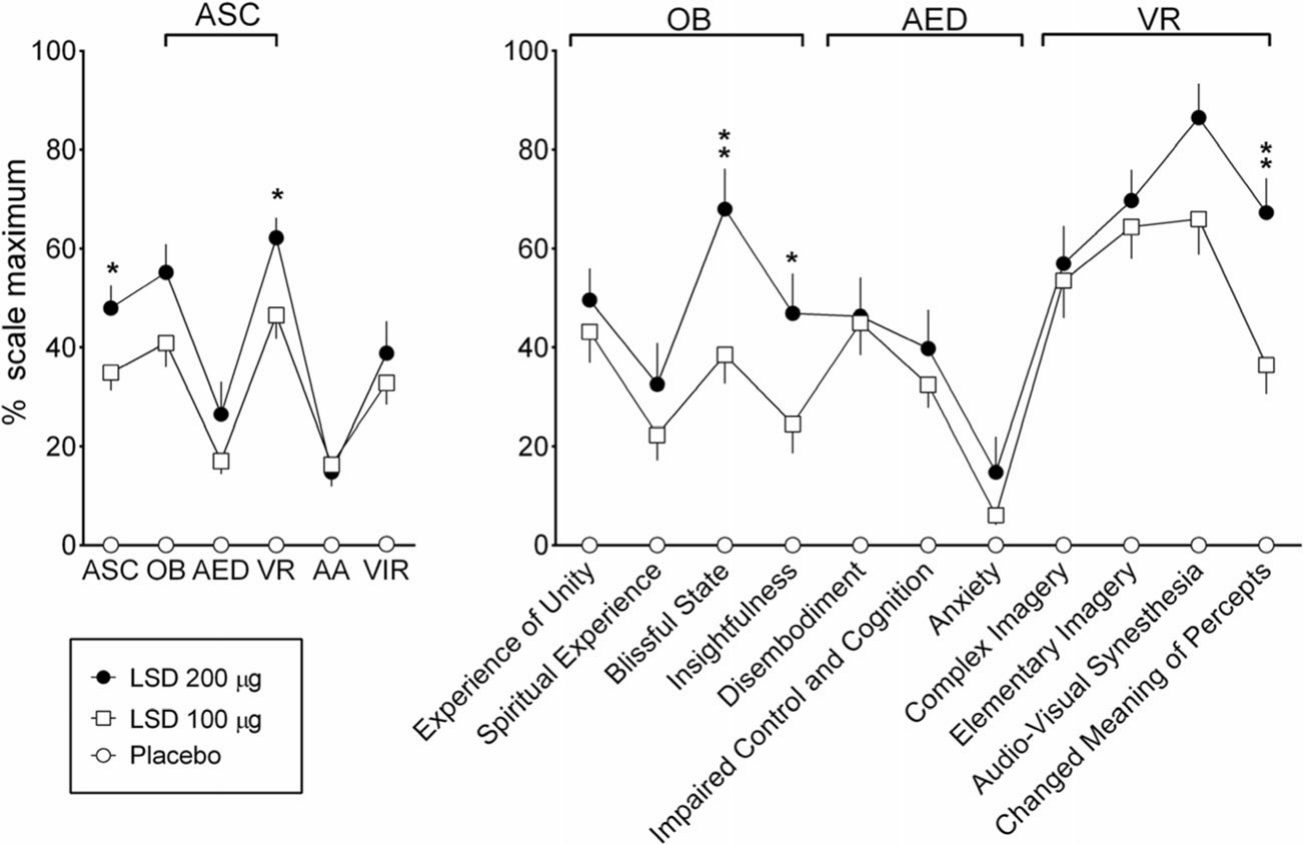
Effects of LSD on the 5 Dimensions of Altered States of Consciousness (5D-ASC) scale. LSD mainly increased ratings of oceanic boundlessness (OB) and visionary restructuralization (VR), with significantly higher ratings for the ASC total score and VR dimension at 200 μg compared with 100 μg. LSD-induced increases in anxious ego dissolution (AED) and auditory alterations (AA) were relatively small. LSD also produced vigilance reduction (VIR). LSD-induced changes on the 5D-ASC scale were significant compared with placebo for both doses and all of the scales, with the exception of the effects of the 200 μg dose on anxiety (Table 1). At 200 μg, LSD produced significant and relevantly higher ratings of blissful state, insightfulness, and changed meaning of percepts compared with 100 μg (*one asterisk p* < 0.05, *two asterisks p* < 0.01, *t* tests). The data are expressed as the mean ± SEM in 24 subjects and 16 subjects for the 100 and 200 μg doses of LSD, respectively

LSD induced pronounced peak alterations of waking consciousness, with significant increases in all dimensions and subscales of the 5D-ASC scale (Fig. 2). The 200 μg dose of LSD produced significantly greater scores on the overall ASC scale, the dimension of visionary restructuralization, and the blissful state, insightfulness, and changed meaning of percepts subscales compared with the 100 μg dose (Fig. 2, Table 1). The mean ± SEM ego dissolution (item 71) scores were 49 ± 6 and 53 ± 10 after the 100 and 200 μg doses, respectively (Table 1). There were only minimal differences between the 5D-ASC ratings at 3, 10, and 24 h (supplementary Fig. S1 online).

### Plasma LSD concentrations

Plasma concentrations varied between subjects, especially at the lower 100 μg dose. The median (range) *C*_max_ values were 1.4 ng/ml (0.32–3.7) and 3.2 ng/ml (1.9–7.1) for the 100 and 200 μg doses, respectively. The corresponding AUC values were 8.5 ng × h/ml (1–19) and 20.7 ng × h/ml (11–39).

### Associations between alterations of consciousness and mystical-type experiences

**Fig. 3:**
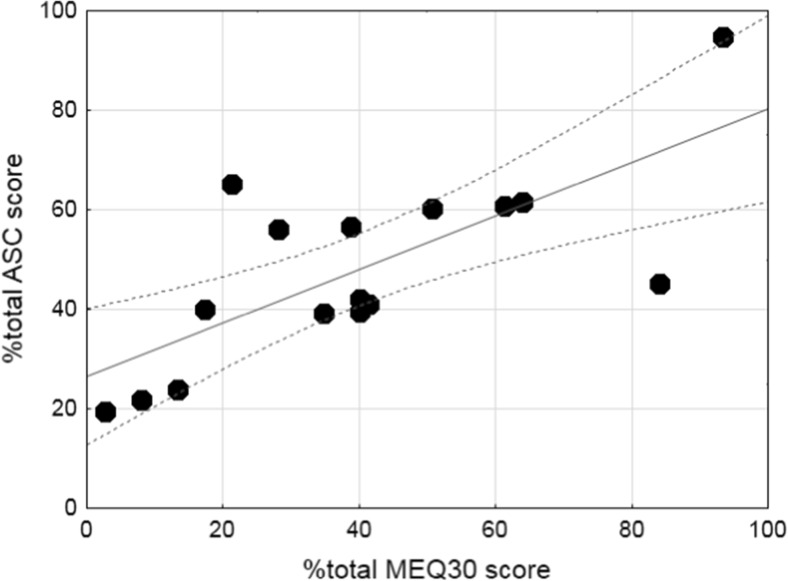
LSD-induced alterations of consciousness are significantly associated with the LSD-induced mystical experience. The data are expressed as a percentage of ASC total scores on the 5D-ASC scale and a percentage of total scores on the MEQ30 for each of 16 participants after administration of 200 μg LSD. The *lines* indicate the regression and 95 % confidence intervals (*R*_*p*_ = 0.87, *p* < 0.001)

Table 2 shows the cross-tabulation of all correlations between the 5D-ASC scale and MEQ30 subscale ratings. LSD-induced alterations of consciousness (ASC total score) were significantly correlated with ratings of mystical experience (MEQ30 total score) on the MEQ (*R*_*p*_ = 0.87, *p* < 0.001, *n* = 16; Fig. 3). Scores on the MEQ positive mood scale were strongly associated with scores on the ASC experience of unity and blissful state scales (*R*_*p*_ = 0.85 and 0.80, respectively; both *p* < 0.001, *n* = 16; Table 2).

**Table 2:** Associations between LSD-induced alterations in consciousness (5D-ASC) and mystical experiences (MEQ30)

	Mystical Effects Questionnaire (MEQ30)				
	MEQ30 total score	Mystical	Positive mood	Transcendence of time/space	Ineffability
5D-ASC scale					
Total ASC score	*0.87*	**0.73**	**0.65**	*0.82*	**0.57**
Oceanic boundlessness	*0.93*	*0.88*	*0.83*	**0.74**	0.45
Anxious ego dissolution	**0.60**	0.39	0.35	**0.68**	**0.55**
Visionary restructuralization	**0.65**	**0.54**	0.38	**0.68**	0.45
Auditory alterations	0.30	0.14	0.02	0.49	0.38
Reductions of vigilance	**0.61**	0.41	0.47	**0.64**	0.47
Experience of unity	*0.82*	*0.86*	*0.85*	**0.56**	0.25
Spiritual experience	*0.79*	*0.76*	*0.76*	**0.60**	0.33
Blissful State	*0.80*	*0.77*	*0.80*	**0.72**	0.16
Insightfulness	*0.77*	*0.79*	**0.68**	**0.52**	0.42
Disembodiment	**0.71**	**0.53**	**0.62**	**0.71**	0.41
Impaired control and cognition	**0.63**	0.37	0.45	*0.79*	0.46
Anxiety	0.45	0.32	0.19	0.47	**0.51**
Complex imagery	0.48	0.31	0.32	**0.69**	0.19
Elementary imagery	0.36	0.37	0.08	0.29	0.42
Audio-visual synesthesia	0.23	0.07	0.22	0.45	−0.01
Changed meaning of percepts	*0.80*	**0.67**	**0.59**	**0.70**	**0.63**
Ego dissolution (item 71)	**0.74**	**0.73**	**0.74**	**0.65**	0.12

Values are Pearson correlation coefficients in 16 subjects describing correlations between %5D-ASC and %MEQ30 scores. Bold values for *P* < 0.05, italic values for *P* < 0.001

### Correlations between plasma LSD concentrations and LSD-induced alterations of consciousness and mystical-type experiences

**Fig. 4:**
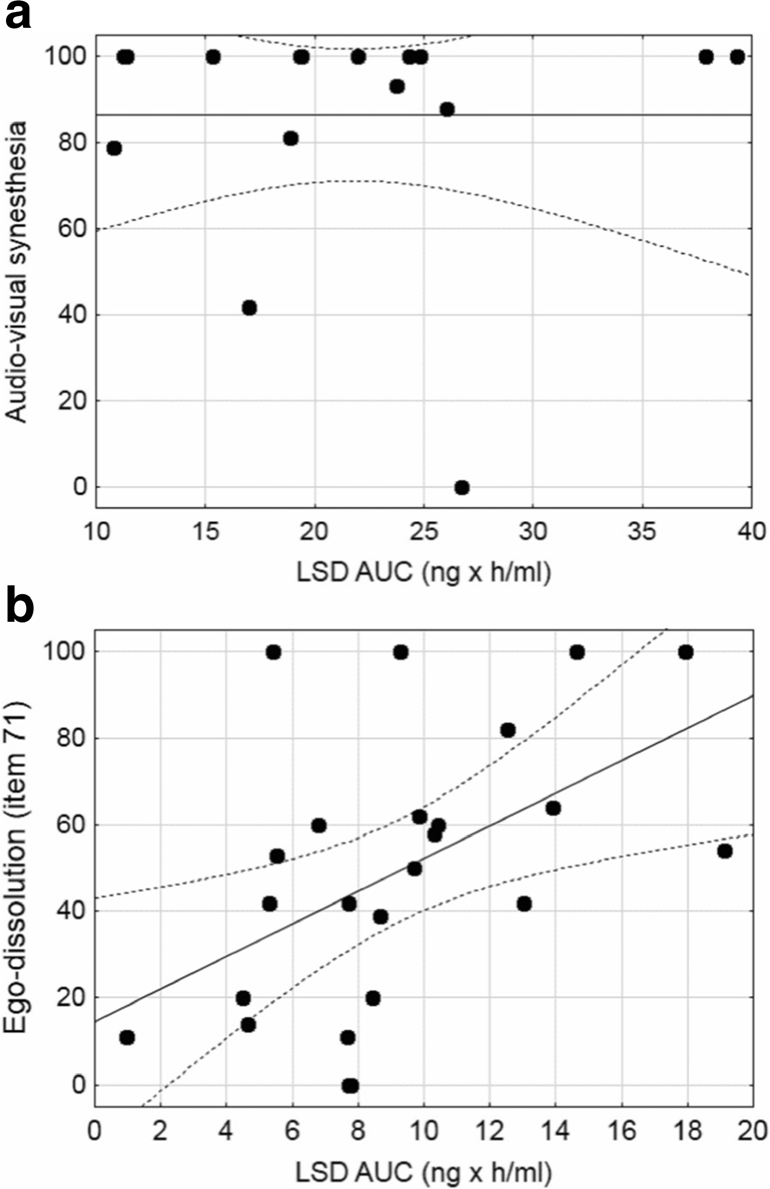
Correlations between plasma LSD concentrations and subjective peak effects. **a** At 200 μg, LSD induced high ratings of audio-visual synesthesia in all but two of the 16 participants. There was little variance in the response and no correlation between total plasma exposure to LSD (area under the concentration-time curve [AUC]) and audio-visual synesthesia (*R*_*p*_ = 0.0, *p* > 0.05, *n* = 16). **b** In contrast, ego dissolution was present to highly variable degrees across subjects after administration of 100 μg LSD. Total exposure to LSD (AUC) positively correlated with LSD-induced ego dissolution (*R*_*p*_ = 0.51, *p* < 0.05, *n* = 24). The *lines* indicate the regression and 95 % confidence intervals

The *C*_max_ and AUC values for LSD were not positively correlated with ratings of peak subjective effects on the 5D-ASC scale or MEQ across subjects or within dose groups (Table 3). For example, LSD induced consistently high ratings of audio-visual synesthesia in almost all of the subjects at the high dose (200 μg), resulting in little within-subject variance and no association with plasma exposure to LSD (Table 3, Fig. 4a). One exception was ego dissolution (item 71) at the lower dose of LSD (100 μg; Table 3, Fig. 4b). The ratings showed high interindividual variance, and there was a significant positive correlation with the LSD AUC value in the 100 μg dose group (*R*_*p*_ = 0.51, *p* < 0.05, *n* = 16; Table 3, Fig. 4b). At the 200 μg dose, there were significant negative correlations between *C*_max_ values for LSD and subjective effects on the 5D-ASC scale including visionary restructuralization, elementary imagery, and changed meaning of percepts.

**Table 3:** Associations between predicted maximal LSD plasma concentrations (*C*_max_) and LSD exposure (AUC) and alterations in consciousness (SD-ASC) and mystical experiences (MEQ30)

	*N* = 24		*N* = 16	
	100 μg		200 μg	
	*C*_max_	AUC	*C*_max_	AUC
5D-ASC scale				
ASC total score	0.19	0.21	−0.35	0.15
Oceanic boundlessness	0.24	0.26	−0.35	0.10
Anxious ego dissolution	0.04	0.07	−0.10	0.32
Visionary restructuralization	0.12	0.15	−**0.59**	−0.16
Auditory alterations	0.02	0.12	−0.18	0.08
Reductions of vigilance	−0.01	0.13	−0.10	0.38
Experience of unity	0.34	0.33	−0.03	0.33
Spiritual experience	−0.02	0.06	−0.32	−0.03
Blissful state	0.25	0.14	−0.23	0.03
Insightfulness	0.24	0.20	−0.37	0.12
Disembodiment	−0.04	0.08	−0.23	0.08
Impaired control and cognition	−0.01	0.01	−0.20	0.18
Anxiety	0.22	0.30	0.01	0.38
Complex imagery	0.06	0.14	−0.28	−0.04
Elementary imagery	−0.13	−0.03	−**0.53**	−0.15
Audio-visual synesthesia	0.23	0.26	−0.01	0.00
Changed meaning of percepts	−0.03	−0.06	−**0.62**	−0.10
Ego dissolution (item 71)	0.40	**0.51**	−0.27	−0.14
MEQ30				
MEC30 total score	NA		−0.30	0.17
Mystical	NA		−0.25	0.13
Positive mood	NA		−0.08	0.21
Transcendence of time/space	NA		−0.23	0.10
Ineffability	NA		−0.49	0.13

Values are Pearson correlation coefficients describing correlations, the peak concentrations of LSD predicted by the one-compartment model, and LSD-induced %5D-ASC and %MEQ30 scores. Bold values for *P* < 0.05*C*_*max*_ maximal LSD plasma concentration predicted by the one-compartment pharmacokinetic model, *AUC* area under the LSD concentration-time curve predicted by the model

## Discussion

The present study characterized LSD-induced mystical experiences using the MEQ after a dose of 200 μg and alterations of consciousness on the 5D-ASC scale after a dose of 100 μg. The study also evaluated associations between plasma LSD concentrations and these subjective effects.

LSD produced mean MEQ30 total score ratings of 61 % (range 40–98 %) and a complete mystical experience in only two participants (12.5 %). The MEQ has typically been used with psilocybin, and data on MEQ30 scores are available for various doses of psilocybin, placebo, and methylphenidate (active placebo; Barrett et al. 2015). Psilocybin (at the highest studied dose of 30 mg/70 kg) produced a high mean MEQ30 total score rating of 77 % and complete mystical experiences in as many as 67 % of healthy subjects (Barrett et al. 2015). However, in this psilocybin study setting, inactive and active placebo (methylphenidate) also produced high mean MEQ30 ratings of 23 and 33 %, respectively (Barrett et al. 2015). In contrast, in the present study, placebo increased MEQ30 scores only to 1 %. Similarly, MDMA and methylphenidate produced only small increases in MEQ scores in a similar laboratory setting (Schmid et al. 2014). Another study evaluated psilocybin-assisted psychotherapy in tobacco smokers and also found complete mystical experiences in only 10 of 26 sessions (38 %) that were conducted in 14 patients with high-dose psilocybin (30 mg/70 kg; Garcia-Romeu et al. 2015; Johnson et al. 2014). Accounting for the higher placebo ratings in some of the psilocybin studies compared with our study, LSD increased MEQ30 score differences from placebo overall more than psilocybin and produced greater ineffability and positive mood but lower effects on the mystical subscale than psilocybin (Barrett et al. 2015).

Additionally, the MEQ has been used in patients with anxiety associated with life-threatening illness who were treated with 200 μg LSD (Gasser et al. 2014; Gasser et al. 2015). In this therapeutic setting, LSD produced similar mystical experiences as in the present study and complete mystical experiences in only two of 11 patients. MEQ scores were only within the range of 3–9 % after active placebo administration (25 μg LSD) on the MEQ subscales. Altogether, these findings indicate that mainly the placebo response and/or the expectancy of a mystical experience were greater in the study setting in some psilocybin studies compared with the LSD studies. Additionally, the participants in the psilocybin studies may have been more spiritually inclined (Griffiths et al. 2006) than our study participants leading to more mystical experiences (Studerus et al. 2012). Furthermore, others may have provided more extensive preparation of the subjects and interpersonal support, contributing to mystical experiences.

The present findings do not support the view that LSD produces lower overall effects than psilocybin at the doses tested. In contrast, the high dose of LSD (200 μg) produced greater placebo-adjusted positive mood ratings than psilocybin on the MEQ30 (Barrett et al. 2015) and very pronounced increases in 5D-ASC blissful state ratings and produced far greater effects than the highest doses of psilocybin or dimethyltryptamine (DMT) that were tested so far on this scale (Gouzoulis-Mayfrank et al. 2005; Hasler et al. 2004). Additionally, LSD-induced MEQ scores were highly correlated with 5D-ASC scores in the present study.

One could argue that mystical and spiritual experiences are not the most prominent feature of the LSD response. Mean ratings on the spiritual experience scale of the 5D-ASC were 22 and 33 % at the 100 and 200 μg doses, respectively, in the present study and approximately 23 % after 75 μg LSD in another study (Carhart-Harris et al. 2016c). Mean ratings of “the experience had a spiritual or mystical quality” were also only approximately 28 % in an imaging study that evaluated the effects of LSD (Tagliazucchi et al. 2016). However, a direct within-subjects comparison of LSD and psilocybin in the same research setting is needed to determine possible differences in mystical-type responses between these substances. Whether mystical-type experiences (Barrett et al. 2015; Garcia-Romeu et al. 2015; MacLean et al. 2011) are critical for the therapeutic potential of substance-assisted psychotherapy requires further study. At least in the case of LSD, the mystical experiences (MEQ scores) were highly associated with other alterations of consciousness on the 5D-ASC scale, and LSD produced additional effects on emotion processing that could facilitate psychotherapeutic interventions (Dolder et al. 2016).

Recent experimental studies associated the subjective effects of LSD (75 μg, intravenous) on the 5D-ASC scale with fMRI data but in the absence of data on plasma LSD levels (Carhart-Harris et al. 2016c; Kaelen et al. 2016; Lebedev et al. 2016; Roseman et al. 2016). Assuming high oral bioavailability of LSD of 70–100 % (Dolder et al. 2015b), similar plasma exposure (AUC) can be assumed after oral administration of 100 μg LSD (present study I) or intravenous administration of 75 μg LSD (all studies by Carhart-Harris and colleagues). Supporting this assumption, the intravenous 75 μg dose of LSD produced very similar mean ratings on the 5D-ASC scale (Carhart-Harris et al. 2016b) to the present study that used an oral dose of 100 μg. In contrast, the 200 μg dose produced significantly greater ASC total scores and particularly greater 5D-ASC subscale scores of blissful state, insightfulness, and changed meaning of percepts. As previously reported, the 200 μg dose of LSD also produced greater feelings of closeness to others, happiness, openness, and trust than the 100 μg dose (Dolder et al. 2016). Altogether, the data indicate that the 200 μg dose produces overall greater effects and particularly more positive and MDMA-like effects than lower doses (Dolder et al. 2016). This is relevant because the higher dose is currently being used in LSD-assisted psychotherapy (Gasser et al. 2014; Gasser et al. 2015), and the lower dose is being tested in experimental fMRI studies (Carhart-Harris et al. 2016c). The 200 μg dose of LSD also produced greater ASC scores than high doses of the serotonergic hallucinogens DMT and psilocybin (Gouzoulis-Mayfrank et al. 2005; Hasler et al. 2004; Vollenweider and Kometer 2010), ketamine (Gouzoulis-Mayfrank et al. 2005; Studerus et al. 2010), and MDMA (Hysek et al. 2011), although direct comparisons within the same studies and subjects are missing.

The present analyses showed no positive correlations between LSD levels and effects across subjects, possibly because of the relatively high levels of LSD and generally consistently high subjective response ratings in most subjects. Thus, if relatively high and similar doses of LSD are used that result in plasma levels clearly above the EC_50_ of a particular response measure, then it is unlikely that the response varies relevantly across subjects because responses are close to maximal. This would typically also be the case with measures with a maximal effect limit such as VAS ratings and some physiological effects like pupil size (Hysek and Liechti 2012).

In fact, responses to MDMA or LSD or other drugs in a standardized experimental setting may vary only if the response is not induced consistently in all subjects (e.g., at the beginning of the response) and are mostly attributable to individual differences in drug absorption/distribution (Hysek and Liechti 2012) or when a response is evaluated that is not robustly induced or when a lower dose is used. Specifically, correlations of plasma levels with the subjective and cardiovascular effects of MDMA across subjects are only weak during the peak response but stronger at onset (Hysek and Liechti 2012). This is an important consideration. For example, LSD-induced subjective ego dissolution was recently shown to be associated with specific brain activation patterns in a study that administered a relatively low dose of LSD of 75 μg intravenously (Tagliazucchi et al. 2016). Interestingly, LSD-induced ego dissolution correlated with plasma LSD levels after administration of an equivalent oral dose of 100 μg in the present study, and this was the only pharmacodynamic effect of LSD for which a positive association with plasma levels could be demonstrated across subjects. This finding needs to be kept in mind when interpreting associations between ego dissolution and fMRI parameters because the fMRI findings may also reflect other processes that are related to the plasma levels of LSD. Furthermore, the likelihood of detecting correlations within a dose group increases for effects that are not robustly induced in all subjects and thus for effects that are not typically present in all subjects after LSD administration. Finally, unclear is the extent to which a full LSD response was induced in the imaging studies that have been conducted to date because all of these studies used relatively low 75 or 100 μg doses. In the present study, the 200 μg dose of LSD produced particularly marked increases in visionary restructuralization including changed meaning of percepts which were significantly greater after the 200 compared with the 100 μg dose. Contrary to expectations, these perceptual alterations were greater in participants with relatively lower *C*_max_ levels of LSD within the 200 μg dose group further supporting the view that higher plasma levels of LSD may not produce greater subjective alterations above a certain threshold level and if high doses of LSD are used.

In conclusion, LSD (200 μg) rarely produced full mystical experiences in the present study and in patients during LSD-assisted psychotherapy compared with psilocybin in another set and setting. This raises questions regarding expectancy effects and placebo responses and the therapeutic role of mystical experiences. LSD produced significantly greater bliss, insightfulness, and changes in meaning of percepts at 200 μg compared with 100 μg, in addition to the previously reported greater empathogenic effects. This could be relevant for LSD-assisted psychotherapy (200 μg) and the interpretation of fMRI data (75–100 μg). Generally, no association was found between plasma LSD levels and its robust effects when analyzed across different subjects and within a dose group. This may have implications for studies that interrelate different effects of LSD, namely fMRI studies.

## Electronic supplementary material

ESM 1 (DOCX 202 kb)

## References

[CR1] Barrett FS, Johnson MW, Griffiths RR (2015). Validation of the revised mystical experience questionnaire in experimental sessions with psilocybin. J Psychopharmacol.

[CR2] Baumeister D, Barnes G, Giaroli G, Tracy D (2014). Classical hallucinogens as antidepressants? A review of pharmacodynamics and putative clinical roles. Ther Adv Psychopharmacol.

[CR3] Bogenschutz MP, Forcehimes AA, Pommy JA, Wilcox CE, Barbosa PC, Strassman RJ (2015). Psilocybin-assisted treatment for alcohol dependence: a proof-of-concept study. J Psychopharmacol.

[CR4] Carhart-Harris RL, Kaelen M, Whalley MG, Bolstridge M, Feilding A, Nutt DJ (2015). LSD enhances suggestibility in healthy volunteers. Psychopharmacology.

[CR5] Carhart-Harris RL, Bolstridge M, Rucker J, Day CM, Erritzoe D, Kaelen M, Bloomfield M, Rickard JA, Forbes B, Feilding A, Taylor D, Pilling S, Curran VH, Nutt DJ (2016). Psilocybin with psychological support for treatment-resistant depression: an open-label feasibility study. Lancet Psychiatry.

[CR6] Carhart-Harris RL, Kaelen M, Bolstridge M, Williams TM, Williams LT, Underwood R, Feilding A, Nutt DJ (2016). The paradoxical psychological effects of lysergic acid diethylamide (LSD). Psychol Med.

[CR7] Carhart-Harris RL, Muthukumaraswamy S, Roseman L, Kaelen M, Droog W, Murphy K, Tagliazucchi E, Schenberg EE, Nest T, Orban C, Leech R, Williams LT, Williams TM, Bolstridge M, Sessa B, McGonigle J, Sereno MI, Nichols D, Hellyer PJ, Hobden P, Evans J, Singh KD, Wise RG, Curran HV, Feilding A, Nutt DJ (2016). Neural correlates of the LSD experience revealed by multimodal neuroimaging. Proc Natl Acad Sci U S A.

[CR8] Davenport WJ (2016). Psychedelic and nonpsychedelic LSD and psilocybin for cluster headache. CMAJ.

[CR9] Diesch MK (2015). LSD: Rückkehr in the Klinische Forschung.

[CR10] Dittrich A (1998). The standardized psychometric assessment of altered states of consciousness (ASCs) in humans. Pharmacopsychiatry.

[CR11] Dolder PC, Liechti ME, Rentsch KM (2015). Development and validation of a rapid turboflow LC-MS/MS method for the quantification of LSD and 2-oxo-3-hydroxy LSD in serum and urine samples of emergency toxicological cases. Anal Bioanal Chem.

[CR12] Dolder PC, Schmid Y, Haschke M, Rentsch KM, Liechti ME (2015b) Pharmacokinetics and concentration-effect relationship of oral LSD in humans. Int J Neuropsychopharmacol 19. doi:10.1093/ijnp/pyv072 10.1093/ijnp/pyv072 PMC4772267 26108222

[CR13] Dolder PC, Schmid Y, Mueller F, Borgwardt S, Liechti ME (2016). LSD acutely impairs fear recognition and enhances emotional empathy and sociality. Neuropsychopharmacology.

[CR14] Garcia-Romeu A, Griffiths RR, Johnson MW (2015). Psilocybin-occasioned mystical experiences in the treatment of tobacco addiction. Curr Drug Abuse Rev.

[CR15] Gasser P, Holstein D, Michel Y, Doblin R, Yazar-Klosinski B, Passie T, Brenneisen R (2014). Safety and efficacy of lysergic acid diethylamide-assisted psychotherapy for anxiety associated with life-threatening diseases. J Nerv Ment Dis.

[CR16] Gasser P, Kirchner K, Passie T (2015). LSD-assisted psychotherapy for anxiety associated with a life-threatening disease: a qualitative study of acute and sustained subjective effects. J Psychopharmacol.

[CR17] Gouzoulis-Mayfrank E, Heekeren K, Neukirch A, Stoll M, Stock C, Obradovic M, Kovar KA (2005). Psychological effects of (S)-ketamine and N,N-dimethyltryptamine (DMT): a double-blind, cross-over study in healthy volunteers. Pharmacopsychiatry.

[CR18] Griffiths R (2016) Overview of the Johns Hopkins psilocybin research project. Interdisciplinary Conference on Psychedelics Research, Amsterdam, June 3–5, 2016

[CR19] Griffiths RR, Richards WA, McCann U, Jesse R (2006). Psilocybin can occasion mystical-type experiences having substantial and sustained personal meaning and spiritual significance. Psychopharmacology.

[CR20] Griffiths R, Richards W, Johnson M, McCann U, Jesse R (2008). Mystical-type experiences occasioned by psilocybin mediate the attribution of personal meaning and spiritual significance 14 months later. J Psychopharmacol.

[CR21] Griffiths RR, Johnson MW, Richards WA, Richards BD, McCann U, Jesse R (2011). Psilocybin occasioned mystical-type experiences: immediate and persisting dose-related effects. Psychopharmacology.

[CR22] Grob CS, Danforth AL, Chopra GS, Hagerty M, McKay CR, Halberstadt AL, Greer GR (2011). Pilot study of psilocybin treatment for anxiety in patients with advanced-stage cancer. Arch Gen Psych.

[CR23] Guss J (2016) The NYU School of Medicine study on psilocybin-assisted therapy for treatment of existential distress in cancer patients: history, study structure, therapist training, outcome data. Interdisciplinary Conference on Psychedelic Research Amsterdam 3–5 June, 2016

[CR24] Hasler F, Grimberg U, Benz MA, Huber T, Vollenweider FX (2004). Acute psychological and physiological effects of psilocybin in healthy humans: a double-blind, placebo-controlled dose-effect study. Psychopharmacology.

[CR25] Hysek CM, Liechti ME (2012). Effects of MDMA alone and after pretreatement with reboxetine, duloxetine, clonidine, carvedilol, and doxazosin on pupillary light reflex. Psychopharmacology.

[CR26] Hysek CM, Simmler LD, Ineichen M, Grouzmann E, Hoener MC, Brenneisen R, Huwyler J, Liechti ME (2011). The norepinephrine transporter inhibitor reboxetine reduces stimulant effects of MDMA ("ecstasy") in humans. Clin Pharmacol Ther.

[CR27] Johnson MW, Garcia-Romeu A, Cosimano MP, Griffiths RR (2014). Pilot study of the 5-HT2AR agonist psilocybin in the treatment of tobacco addiction. J Psychopharmacol.

[CR28] Kaelen M, Barrett FS, Roseman L, Lorenz R, Family N, Bolstridge M, Curran HV, Feilding A, Nutt DJ, Carhart-Harris RL (2015). LSD enhances the emotional response to music. Psychopharmacology.

[CR29] Kaelen M, Roseman L, Kahan J, Santos-Ribeiro A, Orban C, Lorenz R, Barrett FS, Bolstridge M, Williams T, Williams L, Wall MB, Feilding A, Muthukumaraswamy S, Nutt DJ, Carhart-Harris R (2016). LSD modulates music-induced imagery via changes in parahippocampal connectivity. Eur Neuropsychopharmacol.

[CR30] Krebs TS, Johansen PO (2012). Lysergic acid diethylamide (LSD) for alcoholism: meta-analysis of randomized controlled trials. J Psychopharmacol.

[CR31] Krebs TS, Johansen PO (2013). Over 30 million psychedelic users in the United States. F1000Res.

[CR32] Kupferschmidt K (2014). High hopes. Science.

[CR33] Lebedev AV, Kaelen M, Lovden M, Nilsson J, Feilding A, Nutt DJ, Carhart-Harris RL (2016). LSD-induced entropic brain activity predicts subsequent personality change. Hum Brain Mapp.

[CR34] MacLean KA, Johnson MW, Griffiths RR (2011). Mystical experiences occasioned by the hallucinogen psilocybin lead to increases in the personality domain of openness. J Psychopharmacol.

[CR35] MacLean KA, Leoutsakos JM, Johnson MW, Griffiths RR (2012). Factor analysis of the Mystical Experience Questionnaire: a study of experiences occasioned by the hallucinogen psilocybin. J Sci Study Relig.

[CR36] Nichols DE (2016). Psychedelics. Pharmacol Rev.

[CR37] Pahnke WN (1969). Psychedelic drugs and mystical experience. Int Psychiatry Clin.

[CR38] Pahnke WN, Kurland AA, Unger S, Savage C, Grof S (1970). The experimental use of psychedelic (LSD) psychotherapy. JAMA.

[CR39] Passie T, Halpern JH, Stichtenoth DO, Emrich HM, Hintzen A (2008). The pharmacology of lysergic acid diethylamide: a review. CNS Neurosci Ther.

[CR40] Rickli A, Moning OD, Hoener MC, Liechti ME (2016). Receptor interaction profiles of novel psychoactive tryptamines compared with classic hallucinogens. Eur Neuropsychopharmacol.

[CR41] Roseman L, Sereno MI, Leech R, Kaelen M, Orban C, McGonigle J, Feilding A, Nutt DJ, Carhart-Harris RL (2016). LSD alters eyes-closed functional connectivity within the early visual cortex in a retinotopic fashion. Hum Brain Mapp.

[CR42] Schmid Y, Hysek CM, Simmler LD, Crockett MJ, Quednow BB, Liechti ME (2014). Differential effects of MDMA and methylphenidate on social cognition. J Psychopharmacol.

[CR43] Schmid Y, Enzler F, Gasser P, Grouzmann E, Preller KH, Vollenweider FX, Brenneisen R, Muller F, Borgwardt S, Liechti ME (2015). Acute effects of lysergic acid diethylamide in healthy subjects. Biol Psychiatry.

[CR44] Speth J, Speth C, Kaelen M, Schloerscheidt AM, Feilding A, Nutt DJ, Carhart-Harris RL (2016). Decreased mental time travel to the past correlates with default-mode network disintegration under lysergic acid diethylamide. J Psychopharmacol.

[CR45] Steuer AE, Poetzsch M, Stock L, Eisenbeiss L, Schmid Y, Liechti ME, Kraemer T (2016). Development and validation of an ultra-fast and sensitive microflow liquid chromatography-tandem mass spectrometry (MFLC-MS/MS) method for quantification of LSD and its metabolites in plasma and application to a controlled LSD administration study in humans. Drug Test Anal.

[CR46] Strajhar P, Schmid Y, Liakoni E, Dolder PC, Rentsch KM, Kratschmar DV, Odermatt A, Liechti ME (2016). Acute effects of lysergic acid diethylamide on circulating steroid levels in healthy subjects. J Neuroendocrinol.

[CR47] Studerus E, Gamma A, Vollenweider FX (2010). Psychometric evaluation of the altered states of consciousness rating scale (OAV). PLoS One.

[CR48] Studerus E, Gamma A, Kometer M, Vollenweider FX (2012). Prediction of psilocybin response in healthy volunteers. PLoS One.

[CR49] Tagliazucchi E, Roseman L, Kaelen M, Orban C, Muthukumaraswamy SD, Murphy K, Laufs H, Leech R, McGonigle J, Crossley N, Bullmore E, Williams T, Bolstridge M, Feilding A, Nutt DJ, Carhart-Harris R (2016). Increased global functional connectivity correlates with LSD-induced ego dissolution. Curr Biol.

[CR50] Terhune DB, Luke DP, Kaelen M, Bolstridge M, Feilding A, Nutt D, Carhart-Harris R, Ward J (2016). A placebo-controlled investigation of synaesthesia-like experiences under LSD. Neuropsychologia.

[CR51] Turek IS, Soskin RA, Kurland AA (1974). Methylenedioxyamphetamine (MDA)-subjective effects. J Psychoactive Drugs.

[CR52] Vollenweider FX, Kometer M (2010). The neurobiology of psychedelic drugs: implications for the treatment of mood disorders. Nat Rev Neurosci.

[CR53] Vollenweider FX, Csomor PA, Knappe B, Geyer MA, Quednow BB (2007). The effects of the preferential 5-HT_2A_ agonist psilocybin on prepulse inhibition of startle in healthy human volunteers depend on interstimulus interval. Neuropsychopharmacology.

