# Supplementary material for: Alterations of consciousness and mystical-type experiences after acute LSD in humans
Source: Psychopharmacology (Berl). 2016 Oct 7;234(9):1499–510. doi: 10.1007/s00213-016-4453-0 (PMC5420386; doi:10.1007/s00213-016-4453-0)
Supplement: Supplementary file 1 — (DOCX 202 kb) [file 213_2016_4453_MOESM1_ESM.docx]

**Online Supplement:**

**Figure S1. Effects of LSD (100 µg) on the 5 Dimensions of Altered States of Consciousness (5D-ASC) scale repeatedly used at 3, 10, and 24h to retrospectively rate the LSD effects in 24 subjects in Study 1.** The aim of the repeated administration was to test whether self-ratings shortly after the peak response (3h), at the end of the response (10h) or on the next day (at 24h) differ from each other. We hypothesized that there would not be any relevant differences. ANOVA with time as between-subject factor (3, 10, and 24 h) on the total ASC score showed a significant effect of time (F_2,46_=5.50, P>0.01). Tukey post hoc tests showed higher ratings at 3h compared with 10 and 24h (both P<0.01) but no differences between the 10 and 24h ratings. ANOVA with time and dimension (5 main dimensions) as factors showed a significant main effect of time (F_2,46_=6.17, P<0.01) and scale F_4,92_=19.87, P<0.001 and a significant time and dimension interaction (F_8,184_=3.5, P<0.001). Tukey post hoc test showed greater ratings at 3h compared with ratings at 10 h on all dimensions (all P<0.01, Figure S1) and compared with ratings at 24 h for AED, AA, and VIR (all P<0.01). Ratings at 10 h did not differ from ratings at 24h with the exception of ratings for VR which were greater at 24 h compared with 10 h (P<0.05). ANOVA with time and scale (all 11 scales of the 5D-ASC) showed no significant main effect of time (F_2,46_=2.37, P=0.10), a significant main effect of scale (F_10,230_=18.90, P<0.001) and a significant time and scale interaction (F_20,460_=1.81, P<0.05). Post hoc tests showed that only ratings of “impaired control and cognition” were higher at 3h compared with 24h. There were no other differences between the ratings at 3, 10, and 24 h. Together the data indicates higher ratings of the overall effect when assessed during the response at 3h compared to ratings taken immediately after the response or on the next day. However, the differences were minimal and not present between ratings at 10 h and 24 h. OB, oceanic boundlessness; AED, anxious ego-dissolution; VR, visionary restructuralization; AA, auditory alterations; VIR, vigilance reduction. **P<0.01 and ***P<0.001 for 3h vs. 10h; ^++^P<0.01 and ^+++^P<0.001 for 3h vs. 24h; ^#^P<0.05 for 10h vs. 24h (Tukey post hoc tests based on significant time and scale interactions in the ANOVA). The data are expressed as the mean ± SEM in 24 subjects.

**German translation of the Mystical Experience Questionnaire (only MEQ30 items).**

| 1 | Loss of your usual sense of time. Verlust des üblichen Zeitgefühls. | 0 | 1 | 2 | 3 | 4 | 5 |  |
| --- | --- | --- | --- | --- | --- | --- | --- | --- |
| 2 | Experience of amazement. Erfahrung des Erstaunens. | 0 | 1 | 2 | 3 | 4 | 5 |  |
| 3 | Sense that the experience cannot be described adequately in words. Gefühl, dass man die Erfahrung nicht mit Worten angemessen beschreiben kann. | 0 | 1 | 2 | 3 | 4 | 5 |  |
| 4 | Gain of insightful knowledge experienced at an intuitive level. Einsicht und Wissenszugewinn auf einer intuitiven Ebene. | 0 | 1 | 2 | 3 | 4 | 5 |  |
| 5 | Feeling that you experienced eternity or infinity. Gefühl, dass Sie Ewigkeit oder Unendlichkeit erlebten. | 0 | 1 | 2 | 3 | 4 | 5 |  |
| 6 | Experience of oneness or unity with objects and/or persons perceived in your surroundings. Gefühl der Einheit mit Objektien und/oder Personen, die Sie in Ihrer Umgebung wahrnahmen. | 0 | 1 | 2 | 3 | 4 | 5 |  |
| 7 | Loss of your usual sense of space. Verlust des normalen Raumgefühls. | 0 | 1 | 2 | 3 | 4 | 5 |  |
| 8 | Feelings of tenderness and gentleness. Gefühle von Zärtlichkeit und Sanftheit. | 0 | 1 | 2 | 3 | 4 | 5 |  |
| 9 | Certainty of encounter with ultimate reality (in the sense of being able to “know” and “see” what is really real at some point during your experience. Gewissheit zu einer bestimmten Zeit der Sitzung einer ultimativen Wirklichkeit zu begegnen (im Sinn zu "Wissen" und "Sehen" was wirklich real ist). | 0 | 1 | 2 | 3 | 4 | 5 |  |
| 10 | Feeling that you could not do justice to your experience by describing it in words. Das Gefühl, dass Sie der Erfahrung nicht gerecht werden können durch das Beschreiben mit Worten. | 0 | 1 | 2 | 3 | 4 | 5 |  |
| 11 | Loss of usual awareness of where you were. Verlust des Bewusstseins dafür, wo Sie sind. | 0 | 1 | 2 | 3 | 4 | 5 |  |
| 12 | Feelings of peace and tranquility. Gefühl von Friede und Ruhe. | 0 | 1 | 2 | 3 | 4 | 5 |  |
| 13 | Sense of being “outside of” time, beyond past and future. Das Gefühl ausserhalb der Zeit, ausserhalb von Vergangenheit und Zukunft zu sein. | 0 | 1 | 2 | 3 | 4 | 5 |  |
| 14 | Freedom from the limitations of your personal self and feeling a unity or bond with what was felt to be greater than your personal self. Freiheit aus der Begrenzung der eigenen Persönlichkeit und Gefühl der Einheit mit etwas, das grösser war als Sie selber. | 0 | 1 | 2 | 3 | 4 | 5 |  |
| 15 | Sense of being at a spiritual height. Das Gefühl auf einem spirituellen Höhepunkt zu sein. | 0 | 1 | 2 | 3 | 4 | 5 |  |
| 16 | Experience of pure being and pure awareness (beyond the world of sense impressions). Erfahrung des reinen Seins oder des reinen Bewusstseins. | 0 | 1 | 2 | 3 | 4 | 5 |  |
| 17 | Experience of ecstasy. Erfahrung der Ekstase. | 0 | 1 | 2 | 3 | 4 | 5 |  |
| 18 | Experience of the insight that “all is One”. Erfahrung der Einsicht, dass "alles Eins ist". | 0 | 1 | 2 | 3 | 4 | 5 |  |
| 19 | Being in a realm with no space boundaries. In einer Realität sein ohne räumliche Grenzen. | 0 | 1 | 2 | 3 | 4 | 5 |  |
| 20 | Experience of oneness in relation to an “inner world” within. Gefühl der Einheit in Verbindung mit einer inneren Welt. | 0 | 1 | 2 | 3 | 4 | 5 |  |
| 21 | Sense of reverence. Gefühl von Ehrfurcht / Demut. | 0 | 1 | 2 | 3 | 4 | 5 |  |
| 22 | Experience of timelessness. Erfahrung der Zeitlosigkeit. | 0 | 1 | 2 | 3 | 4 | 5 |  |
| 23 | You are convinced now, as you look back on your experience, that in it you encountered ultimate reality (i.e., that you “knew” and “saw” what was really real). Sie sind nun überzeugt wenn Sie auf die Erfahrung zurückblieben, dass Sie der absoluten Realität begegnet sind (das heisst, dass Sie "wissen" und "gesehen haben" was wirklich real ist. | 0 | 1 | 2 | 3 | 4 | 5 |  |
| 24 | Feeling that you experienced something profoundly sacred and holy. Gefühl, dass Sie etwas zutiefst Heiliges erlebt haben. | 0 | 1 | 2 | 3 | 4 | 5 |  |
| 25 | Awareness of the life or living presence in all things. Bewusstsein für das Leben oder die Lebendigkeit in allen Dingen. | 0 | 1 | 2 | 3 | 4 | 5 |  |
| 26 | Experience of the fusion of your personal self into a larger whole. Erfahrung des Eintretens der eigenen Person in ein grösseres Ganzes. | 0 | 1 | 2 | 3 | 4 | 5 |  |
| 27 | Sense of awe or awesomeness. Gefühl der Ehrfurcht / Grossartigkeit. | 0 | 1 | 2 | 3 | 4 | 5 |  |
| 28 | Experience of unity with ultimate reality. Erfahrung der Einheit mit einer Letzten Wahrheit. | 0 | 1 | 2 | 3 | 4 | 5 |  |
| 29 | Feeling that it would be difficult to communicate your own experience to others who have not had similar experiences. Gefühl, dass es schwierig sein wird über die Erfahrung mit jemand zu sprechen, der keine gleichartige Erfahrung gemacht hat. | 0 | 1 | 2 | 3 | 4 | 5 |  |
| 30 | Feelings of joy. Gefühl der Freude. | 0 | 1 | 2 | 3 | 4 | 5 |  |
